# Rare Case of a Combined Cholecystocolonic and Cholecystoduodenal Fistula Presenting With Pneumobilia

**DOI:** 10.1155/2024/1084775

**Published:** 2024-10-04

**Authors:** Fredrick J. Bohanon, Rui-Min D. Mao, Taylor P. Williams, Danny P. Bourgeois, Samuel B. Field, Ravi S. Radhakrishnan, Francisco J. Sanfiel

**Affiliations:** ^1^Lane Regional Medical Center, Lane Surgery Group, 6300 Main Street, Zachary, Louisiana 70791, USA; ^2^Department of Surgery, University of Texas Medical Branch-Galveston, 301 University Boulevard Galveston, Galveston, Texas 77555, USA

**Keywords:** biliary enteric fistulae, cholecystectomy, cholecystocolonic, cholecystoduodenal, cholecystoenteric

## Abstract

**Background:** Cholecystoenteric fistulae are rare complications of gallstone disease, with a reported incidence of 0.5% to 0.9% of cholecystectomies. Cholecystoduodenal is the most common fistula followed by cholecystocolonic fistulae.

**Summary:** We report a case of pneumobilia resulting from a combined cholecystoduodenal and cholecystocolonic fistulae treated with a laparoscopic subtotal cholecystectomy and open repair of the enteric fistulae.

**Conclusion:** Combined cholecystoduodenal and cholecystocolonic fistulae are an extremely rare complication of gallstone disease, and meticulous preoperative planning and operative dexterity are needed to safely manage these unusual fistulae.

## 1. Case Description

Cholecystoenteric fistulae (CEF) have been recognized as a challenging medical condition for at least half a millennium with one of the first descriptions by Peter Paw and Diemer Broeck in 1514 [1]. Charles Murchison [2], Johann Ludwig Wilhelm Thudichum [3], and Ludwig Georg Courvoisier [4] began reporting on the operative discovery and treatment of CEF in the late nineteenth century. Despite the time since first reported, this condition remains a rare and complicated disease to safely identify and treat. CEF is a late complication of cholelithiasis with nondescript symptoms that lead to preoperative identification in only 7.9% of cases [5, 6]. CEF has an incidence ranging from 0.5% to 0.9%, with cholecystoduodenal fistula (CDF) being the most common at approximately 75% [7] while cholecystocolonic fistula (CCF) being significantly less common with 113 reported cases [8]. Combined CDF and CCF is extremely rare with 27 reported cases in the world's literature [2, 6, 9]. Given the rarity of this case and the devastating morbidity or mortality following a misdiagnosis and injury to the gastrointestinal tract, we present a combined new case of CCF and CDF in order to raise awareness to this possible disease process.

**Figure 1:**
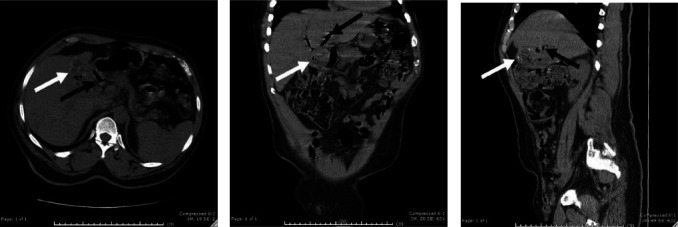
Computerized tomography (CT) images of the abdomen and pelvis without contrast: (A) axial image, (B) coronal image, and (C) sagittal image. White arrow denotes the gallbladder with emphysematous cholecystitis. Black arrow denotes pneumobiila.

The patient is a 62-year-old male with a history of chronic kidney disease, cerebrovascular accident, insulin-dependent diabetes mellitus, hyperlipidemia, hypertension, and a pacemaker in place. The patient presented to the emergency room (ER) for a 1-day history of hiccups, nausea, and vomiting that was unrelenting. In the ER, the patient received Thorazine which resolved the hiccups. He had an abnormal liver function panel with a total bilirubin of 1.7 mg/dL, aspartate transaminase of 70 U/L, alanine transaminase of 205 U/L, and alkaline phosphatase of 276 U/L, and the white blood cell count was 6.4 × 10^9^/L. Computerized tomography (CT) of the abdomen and pelvis without contrast was obtained. The CT revealed significant intra- and extrahepatic pneumobilia and a difficult to identify gallbladder with suspected emphysematous cholecystitis concerning for CEF (Figure 1).

**Figure 2:**
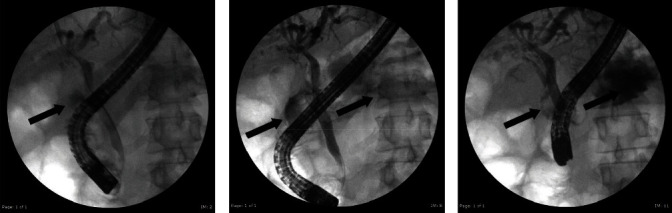
Endoscopic retrograde cholangiopancreatography (ERCP). (A) Occlusion cholangiogram with arrow denoting contrast filling the gallbladder. (B) Arrows denote contrast extravasation from the gallbladder with spillage into the duodenum or stomach. (C) Arrows denote possible cholecystoenteric fistula with contrast pooling in the proximal duodenum/stomach.

The patient was subsequently resuscitated and started on broad-spectrum antibiotics. General surgery and gastroenterology were consulted. A hepatobiliary iminodiacetic acid (HIDA) scan was obtained and demonstrated no uptake by the gallbladder, suggesting acute cholecystitis. Magnetic resonance cholangiopancreatography (MRCP) showed similar findings to that of the CT scan, suggesting a CEF, but no definitive fistula was seen. Endoscopic retrograde cholangiopancreatography (ERCP) was performed by our gastroenterologist. The ERCP illustrated a patent cystic duct with contrast seen in a suspected contracted gallbladder (Figure 2A). Further images revealed contrast extravasation from the gallbladder into the duodenum/stomach (Figure 2B) and a possible fistulous connection to the duodenum (Figure 2C). A sphincterotomy was performed, and biliary stent was placed.

The following day, after informed consent, the patient was taken to the operating room for cholecystectomy and repair of CEF. We began the procedure with the standard four port laparoscopic cholecystectomy technique. Upon entering the abdomen, the gallbladder was opacified by the colon, and after a tedious dissection, we were able to expose the gallbladder fundus. The fundus was opened, and the large stone burden was evacuated allowing for further dissection. Continued dissection of the colon from the gallbladder revealed a small CCF. This fistula was transected sharply, and we proceeded with the dissection. The infundibulum of the gallbladder was densely adherent to the duodenum, and after meticulous dissection, a CDF was identified. This was transected sharply with a LigaSure device. Proximal to this fistula, the tissues were extremely inflamed and fibrotic. We decided at this point to perform a fenestrating subtotal cholecystectomy [10, 11]. We completed the subtotal cholecystectomy and were left with a colotomy and a duodenotomy. We decided to perform these repairs with a conversion to open surgery. The abdomen was opened in the midline, and adequate exposure was obtained. The duodenotomy edges were debrided sharply since we used the LigaSure device to divide the fistula. The duodenotomy was closed transversely with the ECHELON FLEX Powered Vascular Stapler 60-mm blue load. The staple line was then reinforced with 3-0 Vicryl Lembert sutures. The suture line was reinforced with an omental patch. The small colotomy was closed in the same fashion. Patency of the colon and duodenum was confirmed. A Jackson-Pratt (JP) drain was placed within the gallbladder fossa and overlaying the duodenal repair. A feeding gastrojejunostomy tube was placed in the body of the stomach and fed past the duodenal repair well into the proximal jejunum [12]. The abdomen was closed, and the patient was transferred to the recovery room. On postoperative day (POD) 1, the patient had bilious output from his JP; an upper GI tract contrast study was obtained to rule out a duodenal leak. The test confirmed an intact repair with no leak. As expected, the bilious output was from the remaining portion following a subtotal cholecystectomy. The bilious output stopped on POD 2. We began jejunal feeds on POD 3. On POD 6, a clear liquid diet was started, and the patient tolerated this well. The JP drain was removed, and a regular diet was started on POD 8. The patient was able to tolerate PO intake but required ongoing jejunal feeds to supplement caloric intake. The patient was discharged to a skilled nursing facility (SNF) on POD 11. The patient was discharged home from the SNF on POD 16 and was seen in the ER on POD 22 for progressive diarrhea and fatigue. The patient tested positive for *Clostridium difficile* infection and was admitted for resuscitation and treatment. He was discharged on POD 24 with outpatient treatment. The patient was subsequently seen in our office at 1 month and 3 months post-op. The gastrostomy–jejunostomy tube was removed at the first visit. The patient is doing well with no current issues or complaints.

## 2. Discussion

Combined CDF and CCF are an extremely rare finding following gallstone disease and present as a diagnostic and therapeutic dilemma. Patients with CEF have a vast array of symptomatology, from cholangitis to asymptomatic. Our patient presented with nausea, vomiting, and hiccups and was found to have pneumobilia on CT scan of the abdomen, prompting the in-depth investigation to identify the source of the pneumobilia. Preoperative imaging studies may aid in the diagnosis or at least the suggestion of a CEF that will allow for better operative planning. Imaging may include ultrasound, CT scan, HIDA scan, MRCP, and ERCP [13–15]. Traditionally, CEF has been managed with open cholecystectomy, whether planned or forced once identified. Newer studies have demonstrated that laparoscopy may be a safe and feasible option [5, 7, 16]. However, this is a chronic late disease with significant inflammation and scarring that limits the successful completion of the cholecystectomy which is what we encountered. In addition, there is little to be lost by starting laparoscopically. The laparoscopic modality provides outstanding visualization of the operative area that may inform any subsequent open operative maneuvers. Instead of converting to an open cholecystectomy, which newer surgeons have significantly less experience, exposure, and comfort [17], laparoscopic subtotal cholecystectomy is a safe and viable option for the hostile abdomen [10, 11]. Subtotal cholecystectomy was first described by Charles Puestow in 1942 and is now adapted to laparoscopy for an unreceptive right upper quadrant [18]. We were able to successfully take down both fistulous tracts and the vast majority of the gallbladder laparoscopically via the subtotal approach, but we had to convert to an open repair of the duodenotomy. While intracorporeal closure of both colotomy and duodenotomy is in the skill set of an advanced laparoscopic surgeon, the closure of a nonsurgically created duodenotomy is significantly more demanding. The required mobilization and the pliability of inflamed tissues necessitated conversion to an open repair to which we felt more comfortable and safer. To our knowledge, this is the first reported case of a laparoscopic subtotal repair of a CEF.

## 3. Conclusion

CEF is a rare and challenging disease process, more so with a combined CCF and CDF. If CEF is suspected and the patient able to tolerate a workup, significant preoperative planning should be performed with imaging studies to aid in diagnosis and planning. Laparoscopic subtotal cholecystectomy may be a safe treatment option in the newer generation of surgeons with little to no experience in open cholecystectomy and more so in the hostile and unforgiving right upper quadrant.

## Data Availability

The data that support the findings of this study are available on request from the corresponding author. The data are not publicly available due to privacy or ethical restrictions.
